# Estimating Potency in High-Throughput Screening Experiments by Maximizing the Rate of Change in Weighted Shannon Entropy

**DOI:** 10.1038/srep27897

**Published:** 2016-06-15

**Authors:** Keith R. Shockley

**Affiliations:** 1 Biostatistics and Computational Biology Branch, The National Institute of Environmental Health Sciences, National Institutes of Health, 111 T. W. Alexander Drive, Research Triangle Park, NC 27709, USA.

## Abstract

High-throughput *in vitro* screening experiments can be used to generate concentration-response data for large chemical libraries. It is often desirable to estimate the concentration needed to achieve a particular effect, or potency, for each chemical tested in an assay. Potency estimates can be used to directly compare chemical profiles and prioritize compounds for confirmation studies, or employed as input data for prediction modeling and association mapping. The concentration for half-maximal activity derived from the Hill equation model (i.e., *AC*_*50*_) is the most common potency measure applied in pharmacological research and toxicity testing. However, the *AC*_*50*_ parameter is subject to large uncertainty for many concentration-response relationships. In this study we introduce a new measure of potency based on a weighted Shannon entropy measure termed the weighted entropy score (*WES*). Our potency estimator (Point of Departure, *POD*_*WES*_) is defined as the concentration producing the maximum rate of change in weighted entropy along a concentration-response profile. This approach provides a new tool for potency estimation that does not depend on the assumption of monotonicity or any other pre-specified concentration-response relationship. *POD*_*WES*_ estimates potency with greater precision and less bias compared to the conventional *AC*_*50*_ assessed across a range of simulated conditions.

Quantitative high-throughput screening (qHTS) assays^1^ return thousands of concentration-response profiles for large chemical libraries and are currently driving major advancements in drug discovery^2^ and toxicity testing^3^. For example, more than 10,000 substances are now being tested in 15-point concentration-response format in phase II of the Tox21 collaboration, involving the U.S. Environmental Protection Agency (EPA), the U.S. Food and Drug Administration (FDA), the National Institutes of Health (NIH) National Center for Advancing Translational Sciences (NCATS) and the National Institute for Environmental Health Sciences (NIEHS)/National Toxicology Program (NTP)^4^. Response profiles can be summarized by a measure of average activity across tested concentrations, such as the area under the curve (*AUC*) of concentration-response curves^5^, a weighted version of *AUC*^6^, or a weighted entropy score (*WES*)^7^. While these measures are useful for ranking compounds, it is often desirable to estimate the concentration at which a chemical induces a particular effect level using automated data analysis processes. Such potency measures can be applied for rapid identification of pharmacoactive hits or toxicological assessment, or used as input data for prediction modeling^8^ or association mapping^5^.

The most common approach used to approximate chemical potency in chemical genomics and large-scale toxicity testing is the *AC*_*50*_ parameter in the Hill Equation model^9^. The *AC*_*50*_ parameter estimates the concentration at which a chemical produces the half-maximal response along a sigmoidal curve^10^. Incorporating domain knowledge into the curve fitting process can improve agreement between *AC*_*50*_ estimates for sigmoidal curves^11^. However, it is not possible to know the underlying shape of the concentration-response relationship before conducting an experiment^12^ and complex response patterns may reflect real biological responses^13^. Furthermore, linearizing assumptions can render *AC*_*50*_ parameter estimation from the Hill model very unreliable, even with increased sample sizes^10^,^14^. Applying individualized curve fitting procedures can be useful for characterizing screening results. However, in the high-throughput setting manual scrutiny can be restrictively laborious and result in extensive data censoring. Also, while outlier removal and parameter constraints may reduce curve fit error, these procedures do not necessarily increase the repeatability of nonlinear parameter estimation. It is not unusual for *AC*_*50*_ estimates to be accompanied by large standard errors even when one or both asymptotes can be defined^10^.

A point of departure (POD) represents a concentration derived from observed concentration-response data that is associated with a defined effect. *In vitro* POD estimates have been calculated based on linear interpolation between the two concentrations that lie on either side of the assay detection threshold^6^ or establishing a baseline noiseband using the first two tested concentrations^15^. Other POD metrics include an estimate of the concentration producing a predetermined level of an adverse response (i.e., the benchmark dose or BMD) and the highest tested concentration for which there is no observed adverse effect (i.e., the no-observed-adverse-effect-level or NOAEL)^16^,^17^. With true experimental replicates, BMD modeling or NOAEL determinations could serve as POD estimates describing the concentration at which the assay response begins to deviate from baseline response levels. Unlike the NOAEL approach, the BMD procedure uses mathematical modeling to make use of the entire observed concentration-response profile. Unfortunately, in qHTS studies there is usually very little, if any, replication at each tested concentration and it is often not appropriate to combine data across different experimental runs because conditions can change substantially between trials^4^,^10^.

We propose a nonparametric approach based on information theory to improve the precision of compound potency estimation in qHTS studies. Information theoretic concepts were originally developed for communication technology^18^, but these approaches have recently been used to summarize patterns in gene expression microarray data^19^,^20^, find differential methylation sites^21^ and rank chemicals in qHTS experiments^7^. Shannon entropy (*H*) describes the average information content in a probability distribution^22^, and can be used to describe the extent and uniformity of response in a concentration-response profile. Here, *H* is computed from the probability distribution obtained from the observed responses and naturally accommodates any concentration-response pattern, not just monotonic trends such as the sigmoidal shape of the Hill equation model.

We define compound potency as the concentration producing the maximal rate of change in entropy. This potency is calculated by finding the maximum first derivative of the entropy measure across the concentration range. However, Shannon entropy does not take into account the uncertainty in response measurements when responses are within the noise region, i.e., measurements that are less than the assay detection limit. We therefore employ a weighted version of Shannon entropy (or *WES*)^7^. *WES* weights responses found within the noise region so that profiles with larger *WES* scores have greater probability mass (i.e., greater average activity) in the detectable region of the assay. Accordingly, the point of departure is found at the concentration where the rate of change in weighted entropy is maximized along the tested concentration range. This new potency estimator is termed *POD*_*WES*_. Unlike the *AC*_*50*_ value, *POD*_*WES*_ does not rely on the shape of the profile far removed from the point of departure. Observed concentration-response profiles that lie entirely within the assay noise region are assigned the outcome “*undefined*”. Profiles which have detectable responses and for which the maximum rate of change in weighted entropy is located at the lowest observed concentration *C*_*1*_, where *POD*_*WES*_ must be less than *C*_*1*_ but cannot be estimated from the given data, are assigned the outcome “*less than C*_*1*_”.

## Results

### Computing POD_WES_ for illustrative profiles

**Figure 1:**
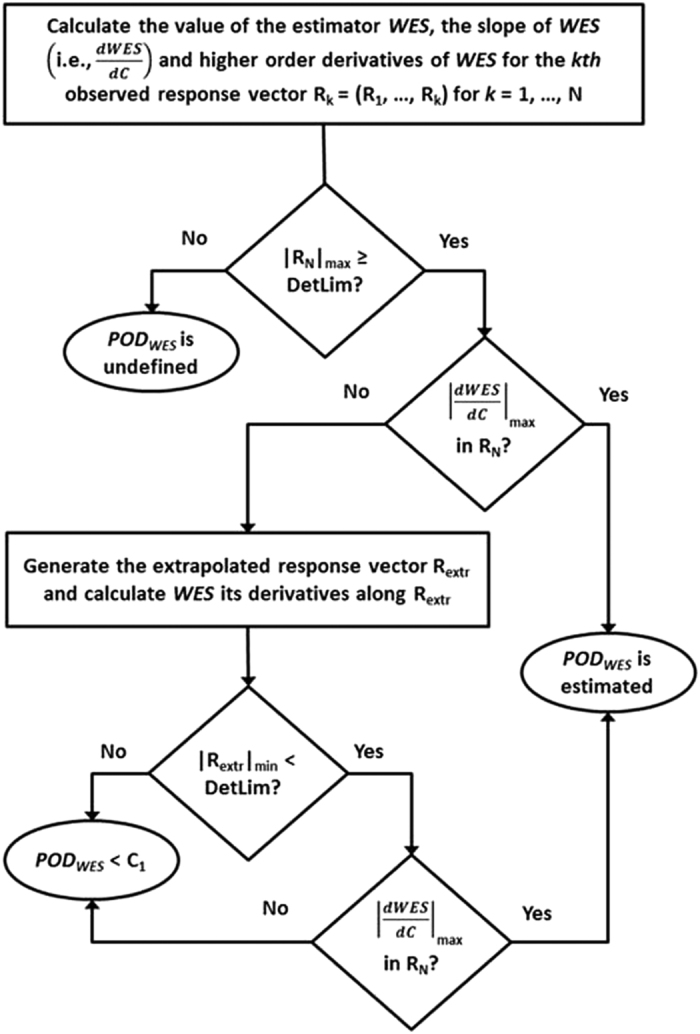
Overview of the potency calculation procedure. *WES* is the weighted entropy score, **R**_k_ is the vector of observed response values (R_1_, …, R_k_), **C**_N_ is the vector of *N* tested concentrations with **R**_N_ observed responses, 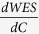 represents the first derivative of *WES* with respect to concentration, and **R**_extr_ is the vector of response values obtained after data extrapolation.

Figure 1 summarizes the workflow used to calculate *POD*_*WES*_. To begin, *WES* and its derivatives are calculated at each tested concentration level. Chemicals with larger *WES* scores have greater average relative responses across concentrations^7^. If the maximum observed response is less than the assay detection limit, *POD*_*WES*_ is “undefined”, since a detectable response may have occurred if a larger range of test concentrations had been used. If at least one measured response is detectable, a search for a maximal rate of change in *WES* is conducted within the observed concentration-response space. If a global 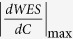 extremum is located, *POD*_*WES*_ is estimated. However, if *POD*_*WES*_ cannot be found, the concentration-response data is extrapolated outside of the observed concentration-response region using finite difference calculus. After extrapolating new responses, *WES* and its derivatives are recalculated and another search for *POD*_*WES*_ is conducted. If *POD*_*WES*_ still cannot be quantitatively determined, but 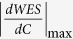 is located at the lowest concentration in the extrapolated profile, *POD*_*WES*_ must be less than the lowest tested concentration (see Supplementary Information).

**Figure 2:**
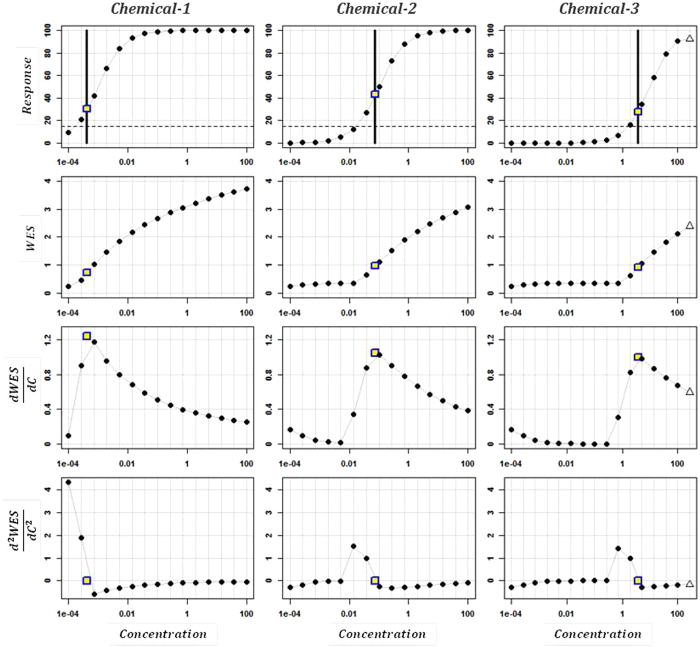
Illustrative example of the proposed approach to calculate potency for three 15-point concentration-response curves generated from the Hill equation model in equation (1) in the Methods with *RMAX* = 100% of the positive control, *R0* = 0, the *h* parameter = 1 and *AC50* = 0.001, 0.1, and 10 for columns 1, 2, and 3, respectively. In the first row, responses are connected by gray lines, where solid circles represent observed responses, an open triangle shows an extrapolated response and dashed lines indicate a detection limit of 15%. Open squares indicate the estimated potency (*POD*_*WES*_). Black vertical bars help to locate the estimated potency on the “Response” graphs. The first row shows the concentration-response, the second row indicates the values of *WES* at each response (i.e., *WES* at concentration *k* is computed by considering only the first 1, …, *k* concentration levels), the third row shows the rate of change in *WES* at each concentration level, and the fourth row indicates the second derivative of *WES* at each concentration level.

Figure 2 depicts hypothetical sigmoidal response profiles for three chemicals. Each chemical follows equation (1) in the Methods with no *ERROR*. The baseline response *R0* is set to 0% of positive control, the maximal response |*RMAX|* is set to 100% of positive control, the *h* parameter is set to 1 and the *AC*_*50*_ is set to 0.001, 0.1, and 10 μM, for *Chemical-1*, *Chemical-2* and *Chemical-3*, respectively. This figure shows the normalized responses (row 1), *WES* computed at each concentration level (row 2), the first derivative of *WES* at each concentration level (row 3) and the second derivative of *WES* at each concentration level (row 4). The concentration at which the first derivative of *WES* is maximized is indicated by an open square.

*Chemical-1* is the most potent of the three chemicals shown in Fig. 2, where only the upper asymptote is well defined. This chemical has a “true” *AC*_*50*_ value equal to 1.00 × 10^−3^ μM, which corresponds to an *POD*_*WES*_ of 4.19 × 10^−4^ μM. *Chemical-2* has two clearly defined asymptotes with an *AC*_*50*_ value of 0.1 μM and a calculated *POD*_*WES*_ of 0.07 μM. One data point, indicated by an open triangle, was extrapolated in order to find *POD*_*WES*_ for *Chemical-3*, which had an *AC*_*50*_ value of 10 μM and a calculated *POD*_*WES*_ of 3.73 μM. In this case, a single extrapolated data point was used in order to calculate the deviation of the estimate of 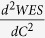 from zero within the prespecified tolerance level (see Supplementary Information for more explanation of the computations). Notice that the value of *WES* at the *kth* concentration level becomes smaller as the *AC*_*50*_ of a profile increases, but the potency measure *POD*_*WES*_ is located at the concentration for which the the rate of change in *WES* is increasing most rapidly. Fig. S1 shows additional examples of *POD*_*WES*_ calculated from curves generated with the “gain-loss” model given in equation (2) in the Methods.

### Evaluating the proposed approach using simulated data

To explore precision and bias of the potency estimates derived from sigmoidal models, we generated 15-point concentration-response profiles from equation (1) in the Methods with *R0* = 0% and *h* = 1 for profiles having [1] only an upper asymptote (*AC*_*50*_ = 0.001 μM), [2] both asymptotes well defined (*AC*_*50*_ = 0.1 μM) and [3] only a lower asymptote (*AC*_*50*_ = 10 μM). In the simulations, *|RMAX|* values were selected as weak (*|RMAX|* = 25%), moderate (*|RMAX|* = 50%), and strong (*|RMAX|* = 100%) activity. A total of 10,000 profiles were generated for each of these nine combinations of *AC*_*50*_ and *|RMAX|* and the residual errors were modeled as *ERROR* ~ N (μ = 0, σ_*i*_^2^) where σ_*i*_ = 5% or σ_*i*_ = 10%. In Table 1, the precision of potency estimation differed markedly between the estimators for the lower error of σ_i_ = 5%. *POD*_*WES*_ estimates were generally more repeatable with confidence interval widths (CIWs) ranging from 1.03 to 1.53 orders of magnitude (OM). *AC*_*50*_ at the same error level ranged from 0.27 to 13.80 OM. The precision of *POD*_*WES*_ at σ_i_ = 10% was comparable to the levels achieved at σ_i_ = 5% for curves simulated under conditions in which “true” maximum response is greater than the detection limit of the assay. By contrast, precision of the *AC*_*50*_ estimator was noticeably lower for σ_i_ = 10% compared to σ_i_ = 5%.

As shown in Table 1, for σ_i_ = 5%, the bias in *POD*_*WES*_ estimates was less than about 2.0-fold on the natural scale, ranging from log_10_Bias = −0.03 (<1.1-fold less than expectation) to log_10_Bias = −0.27 (<1.9-fold less than expectation). For the same data sets, the bias in *AC*_*50*_ estimates ranged from log_10_Bias = −0.0002 (<1.1-fold less than expectation) to log_10_Bias = 1.38 (24.0-fold greater than expected). The estimation bias of *POD*_*WES*_ at σ_i_ = 10% was similar to the values found at σ_i_ = 5%. By contrast, the estimation bias of *AC*_*50*_ was about 10-fold greater for σ_i_ = 10% compared to σ_i_ = 5% in some instances.

In addition to investigating the precision and bias of potency estimators based on the sigmoidal Hill model, we also investigated the precision and bias of estimators using the “gain-loss” model from equation (2) in the Methods. As shown in Table 2, the precision of *POD*_*WES*_ was less than 1.5 OM at σ_i_ = 5% and σ_i_ = 10%. By contrast, the *AC*_*50*_ measure could be extremely imprecise for these curve shapes, even reaching 19.78 OM in one case. Similar to the evaluation of Hill model curves, the log_10_Bias of *POD*_*WES*_ did not exceed −0.42 (<2.7-fold less than expectation). The bias of the *AC*_*50*_ metric was often greater than 2.0 OM (or 100-fold).

### Example compound potency estimates across experimental runs

Previously, the *in vitro* BG1 estrogen receptor alpha (*ERα*) assay from phase II of Tox21 was used to screen for agonist activity in an *ER* reporter gene cell line with an endogenous full length *ERα*. Approximately 10,000 compounds were assayed in three different experimental runs and activity measurements for 15-point concentration-response curves were obtained as luciferase activity readings from the BG1 *ERα* cell line^23^. This data was normalized to 100% of the activity of estrone positive control compounds. Weighted entropy scores (*WES*) and *POD*_*WES*_ values were calculated as described here. Ranking profiles based on *WES* is not based on any pre-specified concentration response model form or direction of response^7^.

**Figure 3:**
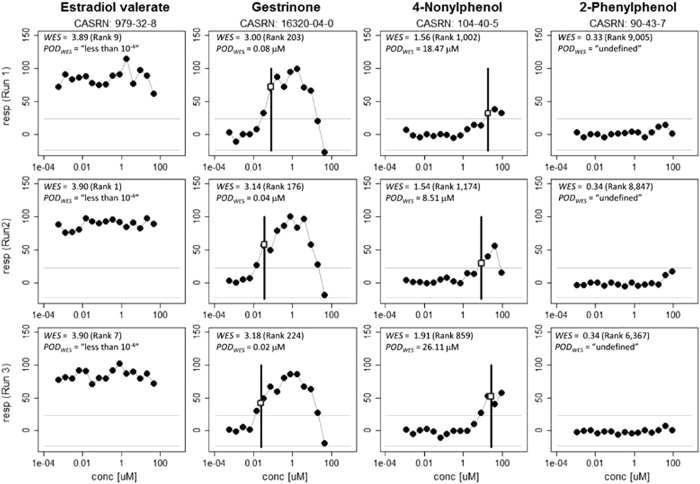
*POD*_*WES*_ values for example concentration-response profiles from the Tox21 Phase II BG1 ER alpha agonist data set. A total of four compounds are shown, with one compound in each column. Each row represents the concentration-response relationship from a separate experimental run. The ranking of each *WES* score out of all 11,776 compounds in the experimental run is given in parentheses. Gray lines indicate the 3σ assay detection limit.

Figure 3 shows concentration-response profiles for four representative compounds that are tested once in each of the three experimental runs. Estradiol valerate is a synthetic ester of the positive control compound 17β-estradiol and is consistently ranked within the top ten compounds based on *WES*. The corresponding potency value for this compound (*POD*_*WES*_) is assigned a value of “less than the lowest tested concentration” in each run. Gestrinone is a synthetic hormone that elicits an agonistic response of 0.05 ± 0.03 μM (across runs) in this experiment, and is ranked in the top 250 compounds based on *WES* in each run shown here. As shown in Fig. 3, the response profile for this compound is better represented by the “gain-loss” model than the Hill model, perhaps due to cytotoxic effects at the greater concentrations. The next compound, 4-Nonylphenol, has previously been shown to act as an agonist of the estrogen receptor alpha in MCF7 breast cancer cells^24^. This compound is ranked within the top 1,200 profiles based on *WES* and has a corresponding *in vitro* potency of 17.7 ± 8.8 μM. Finally, the biocide 2-Phenylphenol does not show detectable activity in the assay in any experimental run and is therefore ranked very low based on the *WES* score in each case. The potency of this compound is assigned the value of “undefined.”

**Figure 4:**
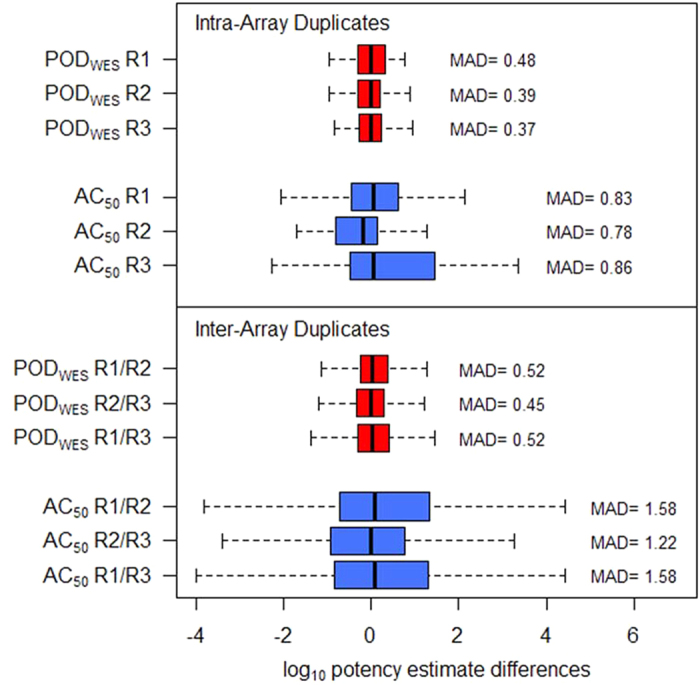
The distribution of log_10_ potency differences (*AC*_*50*_ or *POD*_*WES*_) for intra-array duplicate comparisions (within experimental run R1, R2 and R3) and inter-array duplicate comparisons (between two experimental runs R1/R2, R2/R3 and R1/R3). In the absence of experimental artifacts, it is expected that the log_10_ potency differences would have a median value of zero, which corresponds to a potency ratio of one on the natural scale. The median absolute deviate from zero (*MAD*) is indicated on the figure for each set of log_10_ potency differences.

The reproducibility of the potency estimates in this data set was evaluated by calculating log_10_ potency differences between intra-assay duplicates and inter-assay duplicates interrogated across experimental runs. It is expected that duplicated compounds will have a log_10_ mean ratio of 0, which corresponds to a mean ratio of 1.00 on the natural scale. All duplicated chemicals that had at least one observed response above the assay detection limit were included in the analysis. A shown in Fig. 4, there is less variation in log_10_ potency differences for intra-array duplicates compared to inter-array duplicates as assessed by the median absolute deviation from zero. The dispersion of log_10_ potency differences is noticeably greater for the *AC*_*50*_ value compared to *POD*_*WES*_, indicating that *AC*_*50*_ values are less reproducibile potency estimates in this experiment.

## Discussion

High-throughput screening of compounds for biological activity can play a fundamental role in the advancement of drug discovery^25^ and in efforts to transform toxicology from a mostly observational science into a predictive science^26^. Large-scale qHTS data analyses typically proceed by fitting the Hill equation^9^ to the data and utilizing the *AC*_*50*_ value as an estimate for compound potency. However, the uncertainty (e.g., confidence intervals) of these nonlinear parameter estimates can be extremely large and potentially limit the reproducibility of results obtained from qHTS studies^10^. A new procedure is proposed here to estimate compound potency based on locating the maximum rate of change in weighted entropy. This approach (*POD*_*WES*_) provides more precise estimates of potency than typically obtained by nonlinear parameter estimation from the Hill model.

Regardless of the level of error used to simulate the concentration-response curves, under most circumstances potency measures examined here were subject to empirical confidence interval widths spanning at least one order of magnitude. However, the *CIW* for *AC*_*50*_ estimates extended to greater than 13 orders of magnitude for low efficacy compounds at *|RMAX|* = 25% (see Table 1). Even so, the *CIW* of *POD*_*WES*_ was less than 1.53 orders of magnitude (less than 34-fold) for data simulated from a Hill equation model or a “gain-loss” model. The bias in *POD*_*WES*_ estimates was less than 2.7-fold (or *|log*_*10*_*Bias|* ≤ 0.42), and usually less than 1.5-fold. *AC*_*50*_ estimates showed less bias than *POD*_*WES*_ for Hill model curves generated with two clearly defined asymptotes, but bias was much greater when the data was generated under other conditions.

Across-run comparisons of potency can be more variable than within-run comparisons (see Fig. 4). However, high-throughput screening responses can be affected by both random and systematic error, and run-to-run variability should be not ignored^27^. Obtaining experimental replicates can increase the precision of the potency estimates^28^ and the interpretation of *POD*_*WES*_ may be improved by focusing on robust assays with good agreement between compound measurements^29^ and using appropriate signal curation^6^. If potencies are only desired from a pre-specified functional form (e.g., the Hill model), a two-step procedure can be used to (1) find response profiles that are active according to a robust analysis framework designed to detect the desired trend^30^ and then (2) estimate potencies from the returned profiles.

The repeatability of *AC*_*50*_ estimates can be extremely small for compounds with low efficacy or for situations in which one of the asymptotes cannot be established^10^. Furthermore, the assay detection limit can impact the precision of potency estimation. Using 3σ of the negative controls as a detection limit is a common practice in qHTS studies^1^,^6^,^28^ and the 3σ value performed optimally in our simulation study across a range of σ values when considering bias, precision and the number of profiles with estimable potency values according to the Hill model (Fig. S2) and the “gain-loss” model (Fig. S3). Selective elimination of influential observations will not overcome these issues and may introduce bias because the true concentration-response relationship cannot be known in advance. Difficulties in characterizing the uncertainty of potency estimates derived from pre-specified models may be compounded when response profiles deviate from monotonicity or the incorrect model is employed for nonlinear curve fitting.

Each compound in a qHTS assay can be expected to have a distinctive set of parameters governing its response behavior. However, the approach proposed here to estimate potency using *POD*_*WES*_ does not rely on a pre-specified concentration-response pattern, can be applied to complex response patterns without respect to the direction of response and naturally accommodates missing data into the estimation framework.

## Methods

This section describes the procedures used to estimate compound potency based on maximizing the rate of change in weighted entropy. Data sets are simulated based on the Hill equation in order to evaluate the precision and bias of estimated potencies across a range of parameter space characterizing qHTS studies. In addition, the new potency measure is applied to an experimental data set assaying for estrogen receptor agonist activity from phase II of Tox21.

### Description of simulated data

Similar to previous studies^7^,^10^,^30^, concentration-response data sets were simulated using the logistic form of the four-parameter Hill equation model,


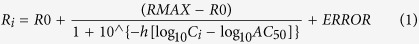


where *R*_*i*_ is a normalized response representing a percentage of the positive control activity at concentration *C*_*i*_. *RMAX* is the maximal response, *R0* is the minimal response, *AC*_*50*_ is the concentration of half-maximal response, *h* affects the shape of the curve and *ERROR* is residual error of the model. The logarithm in equation (1) ensures that back-calculated estimates of *AC*_*50*_ obtained from *log*_*10*_*AC_50_* are restricted to positive values^10^. The concentrations (*C*_*i*_) are based on equivalent log_10_ concentration spacing ranging from 0.0001 to 100 μM for fifteen-point concentration-response curves. The values of *RMAX* and *AC*_*50*_ were set to (25, 50, 100% of positive control activity) and (10^−3^, 10^−1^, 10 μM), respectively, for a total of 9 different data sets. The *R0* parameter was set to 0 and *h* was set to 1. Other data sets were simulated using a “gain-loss” model defined as the product of two Hill equation models,


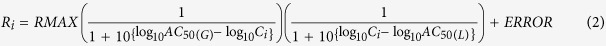


where *RMAX* now represents a shared upper asymptote, both bottom asymptotes equal 0, *AC*_*50(G)*_ is the concentration of half-maximal response in the gain direction and *AC*_*50(L)*_ is the concentration of half-maximal response in the loss direction^15^. Similar to simulations performed using Eqn. (1), the values of *RMAX*, *AC*_*50(G)*_ and *AC*_*50(L)*_ were set to (25, 50, 100%), (10^−3^, 10^−1^, 10 μM) and (10^−3^, 10^−1^, 10 μM), respectively, for a total of 27 different data sets. However, only 12 of these data sets, for which the maximum response (or Peak Response) exceeded the specified detection limit, were included in the analyses here. Residual errors for equations (1) and (2) were modeled as *ERROR* ~ N(0,σ^2^) with σ = 5% or 10%, where σ is related to the percent of negative control responses producing variation levels often seen in Tox21 Phase II assays^6^. Unless otherwise noted, the assay detection limit is taken to be 3σ, a typical detection limit in HTS studies^1^,^6^,^28^. A total of 10,000 simulated substances (*RMAX* = 25%, 50%, or 100% of positive control activity) were simulated for each data set.

### Description of estrogen receptor agonist data set

We acquired qHTS data involving approximately 10,000 compounds that were screened for estrogen receptor alpha agonist activity^23^. This screen utilized an endogenous full length estrogen receptor alpha (BG1 cell line) with a luciferase reporter gene producing a single-channel readout^23^. A total of 15 concentrations were evaluated with concentrations typically ranging from ~10^−3^ μM to ~78 μM. As part of phase II of Tox21, the library is screened three times with compounds located in different well positions during each experimental run^4^. The raw plate reads were normalized using the positive and negative control wells and subsequently corrected for row, column, and plate effects using linear interpolation^23^. Hill equation parameter estimates and activity calls were determined as described previously^30^. In order to assess within-run reproducibility, a set of 88 broadly active duplicates were deliberately included in the Tox21 Phase II 1,536-well assay plates. Concentration-response patterns in this experimental data set encompass many different types of patterns which may deviate substantially from sigmoidal profiles.

### Weighted entropy score

The weighted entropy score provides a measure of average relative activity across a concentration-response profile^7^. Briefly, the response vector for a given substance **R**_**N**_ = (*R*_*1*_, …, *R*_*N*_) contains an observed response *R*_*i*_ for each of *N* concentrations, where *R*_*i*_ corresponds to the response at the *i*th concentration, *C*_*i*_. The relative response at *C*_*i*_ is defined as.


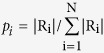


where *p*_*i*_ ≥ 0 and 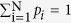. The relative responses *p*_*i*_ define a probability mass distribution based on the magnitude of *R*_*i*_, where *R*_*i*_ may be positive or negative for activation or inhibition, respectively^7^. The entropy of *R*_*i*_, or surprisal of the *ith* event, is defined as


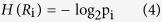


where the units of information are in bits. The weighted average entropy across the response profile takes into account the extent of each response compared to the detection limit of the assay. The weighted entropy score (*WES*) of a substance across *N* concentration levels is given by the expression


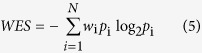


where *WES* ≥ 0 and by convention 0log_2_0 = 0 since 
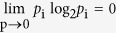
. When every response value is greater than or equal to the assay detection limit, all *w*_*i*_ = 1 so that *WES* is the same as Shannon entropy (i.e., *WES* = *H* = *−*Σ*p*_*i*_log_2_*p*_*i*_). However, when *R*_*i*_ values are less than the assay detection limit of 3σ the weights *w*_*i*_ are defined as the ratio of the surprisal frequency for a relative response within the assay noise region (i.e., −*p*_*i,noise*_log_2_*p*_*i,noise*_, where *p*_*i,noise*_ = *p*_*i*_/3σ) divided by the uncorrected surprisal frequency (i.e., –*p*_*i*_log_2_*p*_*i*_), or *w*_*i*_ = −*p*_*i,noise*_log_2_*p*_*i,noise*_/−*p*_*i*_log_2_*p*_*i*_ = (*p*_i_/*3*σ)log_2_(*p*_i_/*3*σ)*/(p*_*i*_log_2_*p*_*i*_). Larger values of *WES* indicate more detectable responses across concentration levels^7^. The entropy at the *kth* tested concentration (*H*_*k*_ or *WES*_*k*_) is computed by considering only the responses **R**_**k**_ = (*R*_*1*_, …, *R*_*k*_) that are observed within the full concentration-response profile **R**_**N**_.

### The POD Approach to Estimate Potency

We define the profile-specific potency (denoted Point of Departure, POD) as the concentration along the response profile at which the magnitude of the rate of change in *WES* is greatest. This maximum rate of change defines the potency regardless of the direction of change, i.e., irrespective of whether the chemical is an activator or inhibitor. The rate of change in *WES* across the profile is computed as the derivative of *WES* with respect to concentration, or 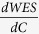, where concentration is based on log_10_ units. In mathematical terms, *POD*_*WES*_ is located at the concentration with the maximal value of 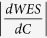 where 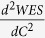 is equal to zero and either (a) 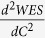 changes sign from positive to negative (for activation) or (b) 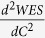 changes sign from negative to positive (for inhibition) according to “The First Derivative Test”^31^. We compute the derivatives of *WES* using finite difference calculus, a mathematical procedure based on a Taylor series procedure that provides difference formulas for a grid sampled at discrete data points^32^. If there are no detectable responses in the profile, the potency is declared “*undefined*”. However, if potency cannot be estimated within the observed response profile but a detectable response is found within data region, finite difference calculus is used to predict the assay response beyond the tested concentration range. This extrapolation continues until *POD*_*WES*_ is quantitatively estimated or designated “*less than C*_*1*_” for profiles that have substantial activity at the lowest observed concentration but no quantitative estimate is obtainable (see Fig. 1). No data points located within the detection window are extrapolated outside of the noise region. The estimation of *POD*_*WES*_ is described in greater detail in the Supplementary Information.

### Evaluating potency estimates

*AC*_*50*_ estimates were determined according to Shockley^30^. The *POD*_*WES*_ approach was described above and presented in Fig. 1. The precision of each potency estimator was investigated by calculating the empirical 95% confidence interval widths (2.5th percentile –97.5th percentile) of the log_10_ transformed estimates within a generated data set. Bias was calculated by subtracting the “true” value *θ* of potency estimator *U* (e.g., *AC*_*50*_ or *POD*_*WES*_ obtained from profiles simulated with ERROR = “0%”) from the mean of the estimated values of *U* according to 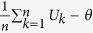. Evaluations of potency estimates are performed using the log_10_ transformation so that the distributions of potencies better approximate a normal distribution with constant error^33^. All computations were performed in the statistical software R^34^.

## Additional Information

**How to cite this article**: Shockley, K. R. Estimating Potency in High-Throughput Screening Experiments by Maximizing the Rate of Change in Weighted Shannon Entropy. *Sci. Rep.* **6**, 27897; doi: 10.1038/srep27897 (2016).

## Supplementary Material

Supplementary Information

## Figures and Tables

**Table 1:** Precision and bias of potency metrics in Hill equation models.

Data Set Parameters			Estimator	
True |*RMAX*|	True *AC*_*50*_	True *POD*_*WES*_	*AC*_*50*_	*POD*_*WES*_
5% error (15% Detection Limit)				
25	1.00e-03	3.17e-03	12.72 (−1.26)	1.26 (+0.11)
25	0.1	0.41	1.59 (−0.001)	1.30 (−0.11)
25	10	43.91	13.80 (+1.38)	1.28 (−0.27)
50	1.00e-03	1.35e-03	3.70 (−0.28)	1.03 (−0.03)
50	0.1	0.18	0.58 (−0.0003)	1.14 (−0.27)
50	10	10.17	2.82 (+0.23)	1.23 (−0.17)
100	1.00e-03	4.19-04	0.62 (−0.03)	1.06 (+0.06)
100	0.1	0.07	0.27 (−0.0002)	1.20 (−0.17)
100	10	3.73	0.63 (+0.02)	1.53 (−0.07)
10% error (30% Detection Limit)				
50	1.00e-03	2.88-03	12.68 (−1.23)	1.34 (+0.11)
50	0.1	0.31	1.53 (+0.002)	1.30 (−0.04)
50	10	39.29	13.67 (+1.40)	1.29 (−0.24)
100	1.00e-03	1.25-03	3.44 (−0.26)	1.00 (−0.03)
100	0.1	0.16	0.57 (−0.001)	0.92 (−0.32)
100	10	9.06	3.79 (+0.27)	0.97 (−0.16)

Equation (1) in the Methods was used to simulate 10,000 fifteen-point concentration-response profiles with the specified error for true values of |*RMAX*| and *AC*_50_, where the hill slope *h* was taken to be 1 and *R*_0_ was specified as 0. Log_10_Precision is shown with log_10_Bias in parentheses. Only data sets with true responses above the assay detection limit were evaluated.

**Table 2:** Precision and bias of potency metrics in gain-loss models.

Data Set Parameters					Estimator	
True |*RMAX*|	True *AC*_*50(G)*_	True *AC*_*50(L)*_	True *POD*_*WES*_	True Peak	*AC*_*50*_	*POD*_*WES*_
5% error (15% Detection Limit)						
25	0.001	0.1	3.23e-03	20.5	2.41 (+2.71)	1.44 (+0.12)
25	0.001	10	3.17e-03	24.5	7.48 (+2.03)	1.32 (+0.11)
25	0.1	10	0.36	20.5	3.28 (−0.82)	1.37 (−0.07)
50	0.001	0.1	1.43e-03	40.95	0.85 (+2.51)	1.03 (−0.04)
50	0.001	10	1.35e-03	49.0	6.42 (+1.95)	1.03 (−0.03)
50	0.1	10	0.18	40.95	0.88 (−0.51)	1.14 (−0.27)
100	0.001	0.001	5.80e-04	24.3	1.32 (+1.15)	0.86 (+0.05)
100	0.001	0.1	4.27e-04	81.9	0.37 (+2.48)	1.07 (+0.05)
100	0.001	10	4.19e-04	98.0	6.44 (+1.98)	1.07 (+0.06)
100	0.1	0.1	0.08	25.0	19.78 (−0.29)	0.95 (−0.20)
100	0.1	10	0.07	81.9	0.37 (−0.48)	1.21 (−0.16)
100	10	10	9.47	24.3	1.36 (−1.17)	0.94 (−0.42)
10% error (30% Detection Limit)						
50	0.001	0.1	2.55e-03	40.95	2.60 (+2.72)	1.44 (+0.17)
50	0.001	10	2.89e-03	49.0	7.33 (+1.96)	1.32 (+0.12)
50	0.1	10	0.31	40.95	3.47 (−0.80)	1.36 (−0.04)
100	0.001	0.1	1.37e-03	81.9	0.87 (+2.51)	1.00 (−0.06)
100	0.001	10	1.25e-03	98.0	6.42 (+1.95)	1.00 (−0.03)
100	0.1	10	0.16	81.9	0.87 (−0.51)	0.94 (−0.32)

Equation (2) in the Methods was used to simulate data sets with the specified error. Log_10_Precision is shown with log_10_Bias in parentheses. Only data sets with true responses above the assay detection limit were evaluated.
