# Supplementary material for: Estimating Potency in High-Throughput Screening Experiments by Maximizing the Rate of Change in Weighted Shannon Entropy
Source: Sci Rep. 2016 Jun 15;6:27897. doi: 10.1038/srep27897 (PMC4908415; doi:10.1038/srep27897)
Supplement: Supplementary Information [file srep27897-s1.pdf]

# Supplementary Information

## Estimating Potency in High-Throughput Screening Experiments by Maximizing the Rate of Change in Weighted Shannon Entropy

Keith R. Shockley

Biostatistics and Computational Biology Branch, The National Institute of Environmental Health Sciences, National Institutes of Health, Research Triangle Park, NC, USA

***Calculating Derivatives using Finite Difference Calculus.*** Following Lynch (2005)[1], consider a function $U$ that is sampled at concentrations $C_1$, .., $C_N$. Here, $U_i$ represents the value of $U$ at concentration $i$, where $U$ can be the *WES* statistic described previously[2]. As shown in Figure S4, a concentration-response grid system corresponding to forward, backward, or central differences can be constructed where a vector **x** ($x_0$, …, $x_i$) quantifies distances between consecutive concentrations. Then:

$$U_i = U_0 + \sum_{n=1}^{\infty} \frac{1}{n!} \frac{d^n U_0}{dC^n} x_i^n \quad (5)$$

Weights ($W_i$) can be invented and applied to equation (5), where $i$ runs across all grid points, as shown below:

$$\sum_i W_i U_i = U_0 \sum_i W_i + \sum_{n=1}^{\infty} \frac{1}{n!} \frac{d^n U_0}{dC^n} \sum_i W_i x_i^n \quad (6)$$

Equation (6) can be simplified for a finite value of $n$ so that the calculation of $U_i$ (for data extrapolation) or a derivative of $U_0$ (for the moments of estimator $U$) can be approximated with the leading error accurate to the order of $(x_0)^2$, where $|x_0| = |L|$ is the distance between the concentration at the origin of the grid and the first concentration point considered in Figure S4. The system of equations in equation (6) can be written as **Aw** = **b**, where **w** is the vector of weights ($W_i$) that can be calculated using the solve() function in the statistical programming environment R[3]. In this manner, equation (6) can be used to calculate the *nth* derivative $\frac{d^n U_0}{dC^n}$ for $n$ = 1, …, 5, each of which are accurate according to $O(L^2)$. The finite difference scheme (i.e., the grid system to use for each calculation) selected to correspond to leading errors of $O(L^2)$ for

each derivative and concentration level $C_1$, …, $C_L$ is given in Table S1. It should be noted that polynomials and splines may be used for interpolation. However, some functions are not well approximated by polynomials and interpolation using high degree polynomials can be unreliable[4,5]. Splines are a useful approach to fit a smooth curve through observed data. However, such techniques are not always mathematically appropriate for extrapolation beyond the range of the data. We employ finite difference calculus for interpolation and extrapolation of data points along the concentration-response profile subject to the constraints of equation (6).

***Example Application of the Finite Difference Approach.*** To illustrate this procedure, consider the calculation of the second derivative of the *WES* score $\left(\frac{d^2WES}{dC^2}\right)$ at the first concentration ($C_1$). In order to approximate the second derivative of *WES* with the leading error of order of $L^2$, it is necessary to use forward differences (see Table S1), where from equation (5) we have

$$\begin{Bmatrix} WES_{i+1} \\ WES_{i+2} \\ WES_{i+3} \end{Bmatrix} = WES_i \begin{Bmatrix} 1 \\ 1 \\ 1 \end{Bmatrix} + \frac{L}{1!}\frac{dWES_i}{dC}\begin{Bmatrix} 1 \\ \alpha+1 \\ \alpha+1+\beta \end{Bmatrix} + \frac{L^2}{2!}\frac{d^2WES_i}{dC^2}\begin{Bmatrix} 1 \\ (\alpha+1)^2 \\ (\alpha+1+\beta)^2 \end{Bmatrix}$$

$$+ \frac{L^3}{3!}\frac{d^3WES_i}{dC^3}\begin{Bmatrix} 1 \\ (\alpha+1)^3 \\ (\alpha+1+\beta)^3 \end{Bmatrix} + \frac{L^4}{4!}\frac{d^4WES_i}{dC^4}\begin{Bmatrix} 1 \\ (\alpha+1)^4 \\ (\alpha+1+\beta)^4 \end{Bmatrix} + \cdots \quad (7)$$

Given a concentration-response vector, *WES* can be calculated at any given point in the response profile (see the discussion in the Methods section). Since the concentrations are known, the values of $L$, $\alpha$ and $\beta$ can be calculated for the appropriate grid system (see Figure S4). However, the derivatives of *WES* in equation (7) need to be determined. Equation (7) can be expressed as a weighted sum of the equations as described by equation (6), where the first weight is arbitrary since the result can be multiplied by a constant[1]. Therefore, we have:

$$WES_{i+1} + W_1 * WES_{i+2} + W_2 * WES_{i+3}$$

$$= WES_i(1 + W_1 + W_2) + L\frac{dWES_i}{dC}(1 + (\alpha + 1)W_1 + (\alpha + 1 + \beta)W_2)$$

$$+ \frac{L^2}{2!}\frac{d^2WES_i}{dC^2}(1 + (\alpha + 1)^2W_1 + (\alpha + 1 + \beta)^2W_2)$$

$$+ \frac{L^3}{3!}\frac{d^3WES_i}{dC^3}(1 + (\alpha + 1)^3W_1 + (\alpha + 1 + \beta)^3W_2)$$

$$+ \frac{L^4}{4!}\frac{d^4WES_i}{dC^4}(1 + (\alpha + 1)^4W_1 + (\alpha + 1 + \beta)^4W_2) + \ \dots \qquad (8)$$

Setting the 1st and 3rd moments equal to zero in equation (8) and rearranging terms, we obtain:

$$\frac{d^2WES_i}{d^2C} = \frac{2\left(WES_{i+1} + W_1 * WES_{i+2} + W_2 * WES_{i+3} - \ WES_i(1 + W_1 + W_2)\right)}{L^2(1 + (\alpha + 1)^2W_1 + (\alpha + 1 + \beta)^2W_2)} + O(L^2) \quad (9)$$

Equation (9) is an expression for the 2nd order derivative of the weighted entropy score where only $W_1$ and $W_2$ are unknown. Setting the *1st* and *3rd* order moments of equation (8) equal to zero provides two equations and two unknowns ($W_1$ and $W_2$):

$$1 + (\alpha + 1)W_1 + (\alpha + 1 + \beta)W_2 = 0 \qquad (10)$$

$$1 + (\alpha + 1)^3W_1 + (\alpha + 1 + \beta)^3W_2 = 0 \qquad (11)$$

Equations (10) and (11) constitute a system of linear equations which can be represented in the algebraic notation of **Aw** = **b**, where

$$\mathbf{A} = \begin{bmatrix} (\alpha + 1) & (\alpha + 1 + \beta) \\ (\alpha + 1)^3 & (\alpha + 1 + \beta)^3 \end{bmatrix} \qquad (12)$$

$$\mathbf{w} = \begin{bmatrix} W_1 \\ W_2 \end{bmatrix} \qquad (13)$$

$$\mathbf{b} = \begin{bmatrix} -1 \\ -1 \end{bmatrix} \tag{14}$$

Solving for **w** can be accomplished using the solve() function in R[3]. In this example, the solution for the components of **w** can be simplified to:

$$W_1 = \frac{1 - (1 + \alpha + \beta)^2}{((1 + \alpha + \beta)^2 - (1 + \alpha)^2)(1 + \alpha)}$$

$$W_2 = \frac{(1 + \alpha)^2 - 1}{(1 + \alpha + \beta)^3 - (1 + \alpha)^2(1 + \alpha + \beta)}$$

***Calculating $POD_{WES}$.*** The estimated concentration for which the second derivative of the *WES* score is zero needs to be calculated in order to determine $POD_{WES}$, the concentration corresponding to $\left|\frac{dWES}{dC}\right|_{max}$ (see Figure 1 in main text). $POD_{WES}$ is located for the maximal value of $\left|\frac{dWES}{dC}\right|$ where $\frac{d^2WES}{dC^2}$ is equal to zero and $\frac{d^2WES}{dC^2}$ changes sign from positive to negative (for activation) or $\frac{d^2WES}{dC^2}$ changes sign from negative to positive (for inhibition) according to "The First Derivative Test"[6]. This concentration can be estimated from the Taylor series expansion of *WES* evaluated at the observed concentration producing $\left|\frac{dWES}{dC}\right|_{max}$, or $POD_{WES,obs}$. For ease of presentation, let $POT = POD_{WES}$ and $POT_{obs} = POD_{WES,obs}$,

$$WES(x) = \sum_{n=0}^{\infty} \frac{\left.\frac{d^nWES}{dC^n}\right|_{x_{obs}}}{n!}(POT - POT_{obs})^n \tag{15}$$

Using the 5th Taylor expansion of equation (15), the second derivative approximation is,

$$\left.\frac{d^2WES}{dC^2}\right|_x = \left.\frac{d^2WES}{dC^2}\right|_{x_{obs}} + \left.\frac{d^3WES}{dC^3}\right|_{x_{obs}} (POT - POT_{obs}) + \left.\frac{d^4WES}{dC^4}\right|_{x_{obs}} (POT - POT_{obs})^2 + \left.\frac{d^5WES}{dC^5}\right|_{x_{obs}} (POT - POT_{obs})^3 \quad (16)$$

The value of *POT* can be found by setting equation (16) equal to zero and using the optimize() function in R[3].

***Description of the Extrapolation Process.*** Extrapolation beyond the observed concentration-response region only occurs if there are detectable responses in a profile and a potency estimate cannot be determined within the observed concentration-response region. To extrapolate below the lowest tested concentration, forward differences are used. A quantity *L* can be defined as the distance between the lowest observed concentration $C_1$ and the second lowest observed concentration $C_2$ as shown in Figure S4. A response value can be extrapolated at a concentration *L* units lower than the lowest tested concentration $C_1$ using the finite difference approach. This extrapolation continues until $POD_{WES}$ can be estimated (the deviation of the estimated second derivative from zero must be less than a prespecified tolerance, which by default this value is set to $10^{-2}$) or the algorithm exceeds its iteration tolerance (i.e., when $\frac{d^2Resp}{dC^2}$ does not change sign in the extrapolation region or no observed responses exceeding the detection limit can be used in the extrapolation process). If the low-concentration extrapolation algorithm reaches its iteration tolerance without convergence, $POD_{WES}$ is assigned the outcome "*less than* $C_1$". This situation could arise under certain circumstances in when there is detectable activity at the lowest tested concentration. To extrapolate above the highest tested concentration, a concentration is selected *L* units ($L = C_N - C_{N-1}$) greater than the highest tested concentration using forward differences.

All the observed and extrapolated data are combined into a single profile and $POD_{WES}$ estimated as described above. A second set of extrapolations is conducted if $\left|\frac{dWES}{dC}\right|_{max}$ is still located at the first or last concentration in the response vector resulting from the first extrapolation process. The $POD_{WES}$ estimate from the first extrapolation is used if this estimate corresponds to a greater $\left|\frac{dWES}{dC}\right|_{max}$ than the one obtained after the second extrapolation.

**Supplemental Figure S1.** Illustrative example of the proposed approach to calculate potency for three 15-point concentration-response curves generated from the "gain-loss" model in equation (2) of the main text with *RMAX* = 100% of the positive control, *AC50(L)* = 10 and *AC50(G)* = 0.001, 0.1, and 10 for columns 1, 2, and 3, respectively. In the first row, responses are connected by gray lines, where solid circles represent observed responses, open triangles indicate extrapolated responses and the detection limit was set at 15%. Squares indicate the estimated potency ($POD_{WES}$). Black vertical bars help to locate the potency on the "Response" graphs. The first row shows the concentration-response, the second row indicates the values of *WES* at each response (i.e., *WES* at concentration *k* is computed by considering only the first 1, …, *k* concentration levels), the third row shows the rate of change in *WES* at each concentration level, and the fourth row indicates the second derivative of *WES* at each concentration level.

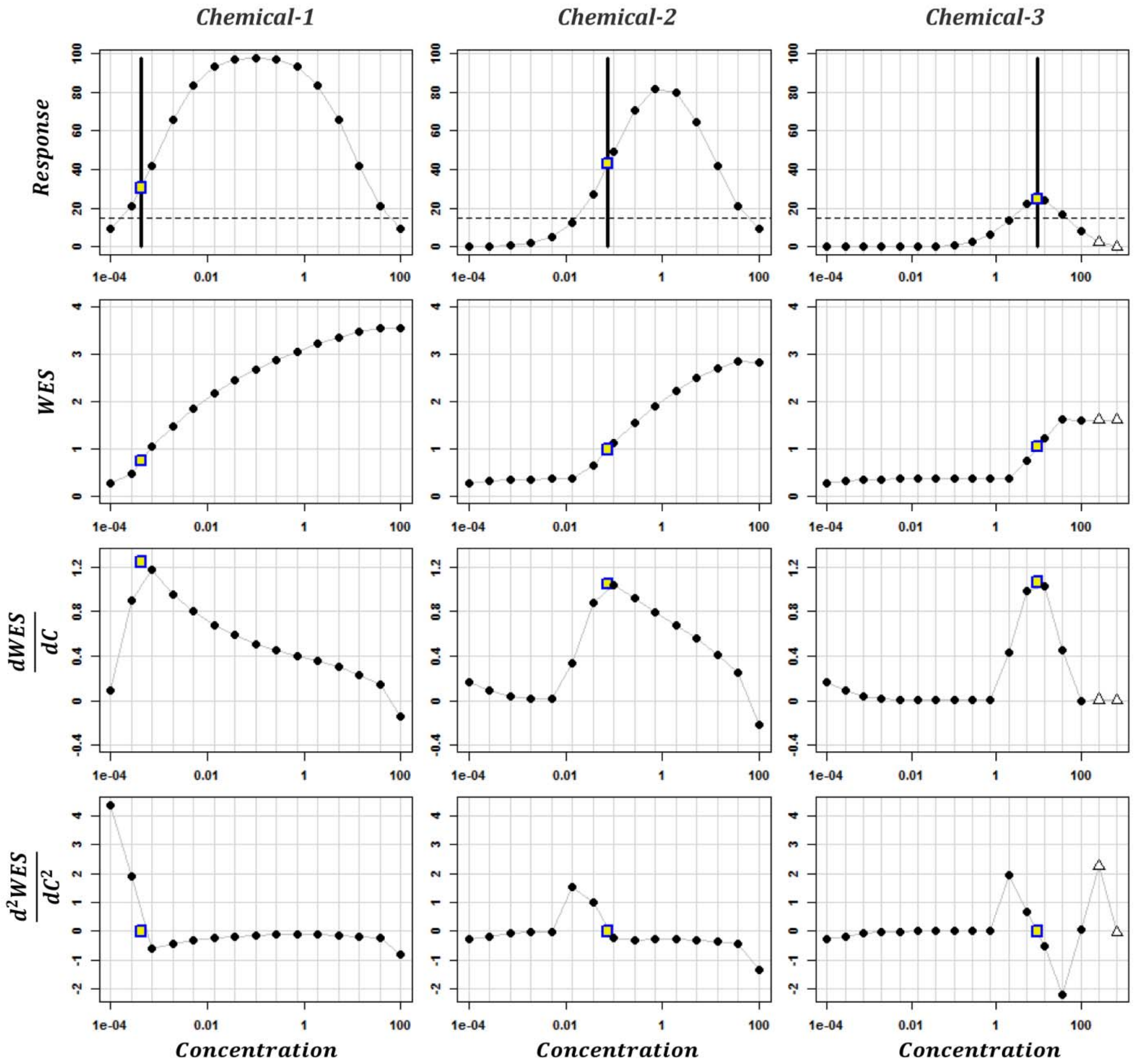

**Supplemental Figure S2.** The calculation of $POD_{WES}$ in data sets simulated from the equation (1) in the main text with 5% error depends on the assay detection limit, which in turn depends on the standard deviation (σ) of the negative controls. Distribution of (A) bias in $POD_{WES}$, (B) precision of $POD_{WES}$ and (C) the number of profiles with an estimated $POD_{WES}$ from 90,000 simulated profiles (15-point concentration-response curves) is presented as a function of σ which is used to define the assay detection limit. The bias is calculated as $\frac{1}{n}\sum_{k=1}^{n} POD_{WES} - \theta$, and the precision is presented as the empirical 95% confidence interval width (*CIW*) of the estimated $POD_{WES}$ as described in the Methods section of the main text.

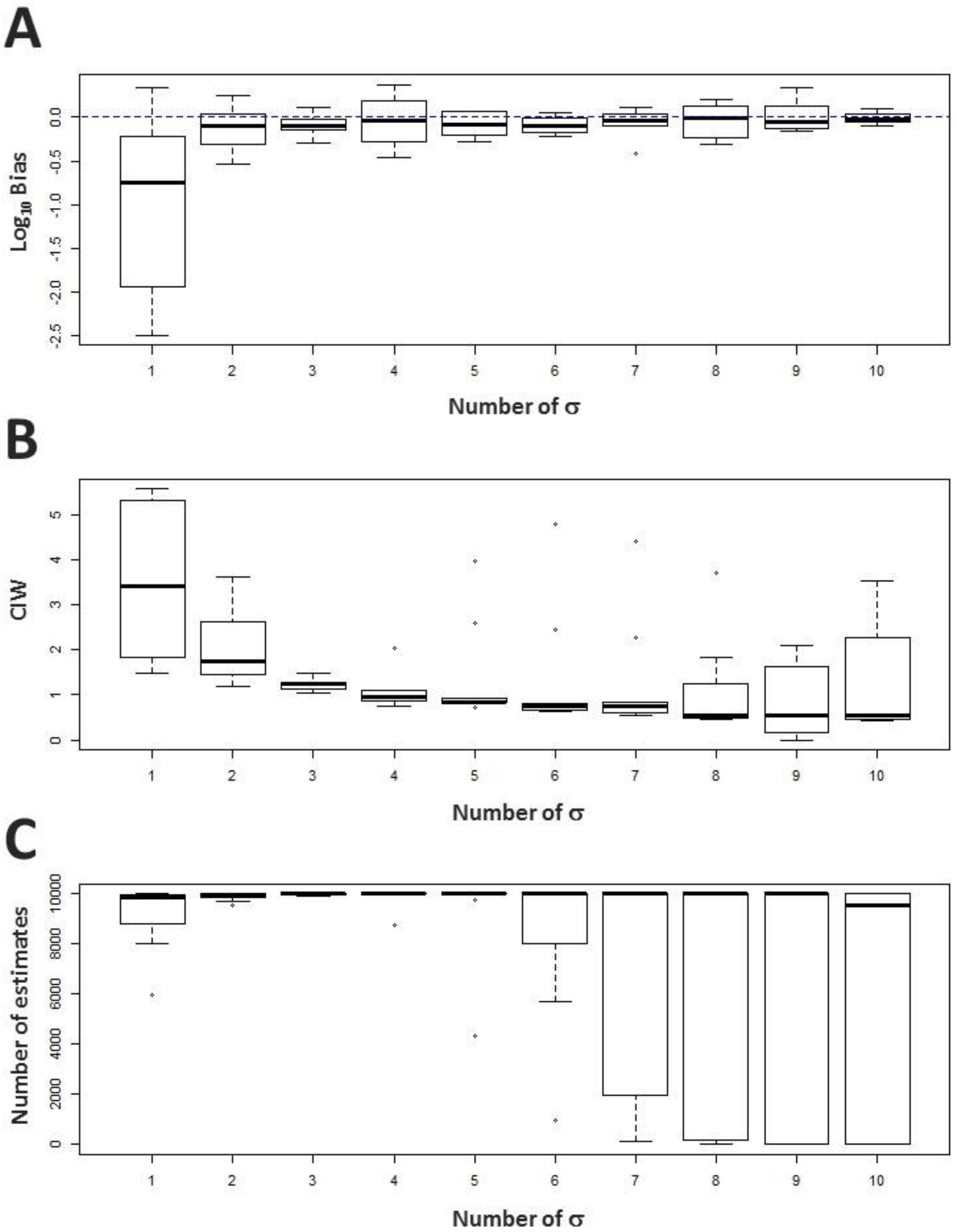

**Supplemental Figure S3.** The calculation of $POD_{WES}$ in data sets simulated from the equation (2) in the main text with 5% error depends on the assay detection limit, which in turn depends on the standard deviation (σ) of the negative controls. Distribution of (A) bias in $POD_{WES}$, (B) precision of $POD_{WES}$ and (C) the number of profiles with an estimated $POD_{WES}$ from 90,000 simulated profiles (15-point concentration-response curves) is presented as a function of σ which is used to define the assay detection limit. The bias is calculated as $\frac{1}{n}\sum_{k=1}^{n} POD_{WES} - \theta$, and the precision is presented as the empirical 95% confidence interval width (*CIW*) of the estimated $POD_{WES}$ as described in the Methods section of the main text.

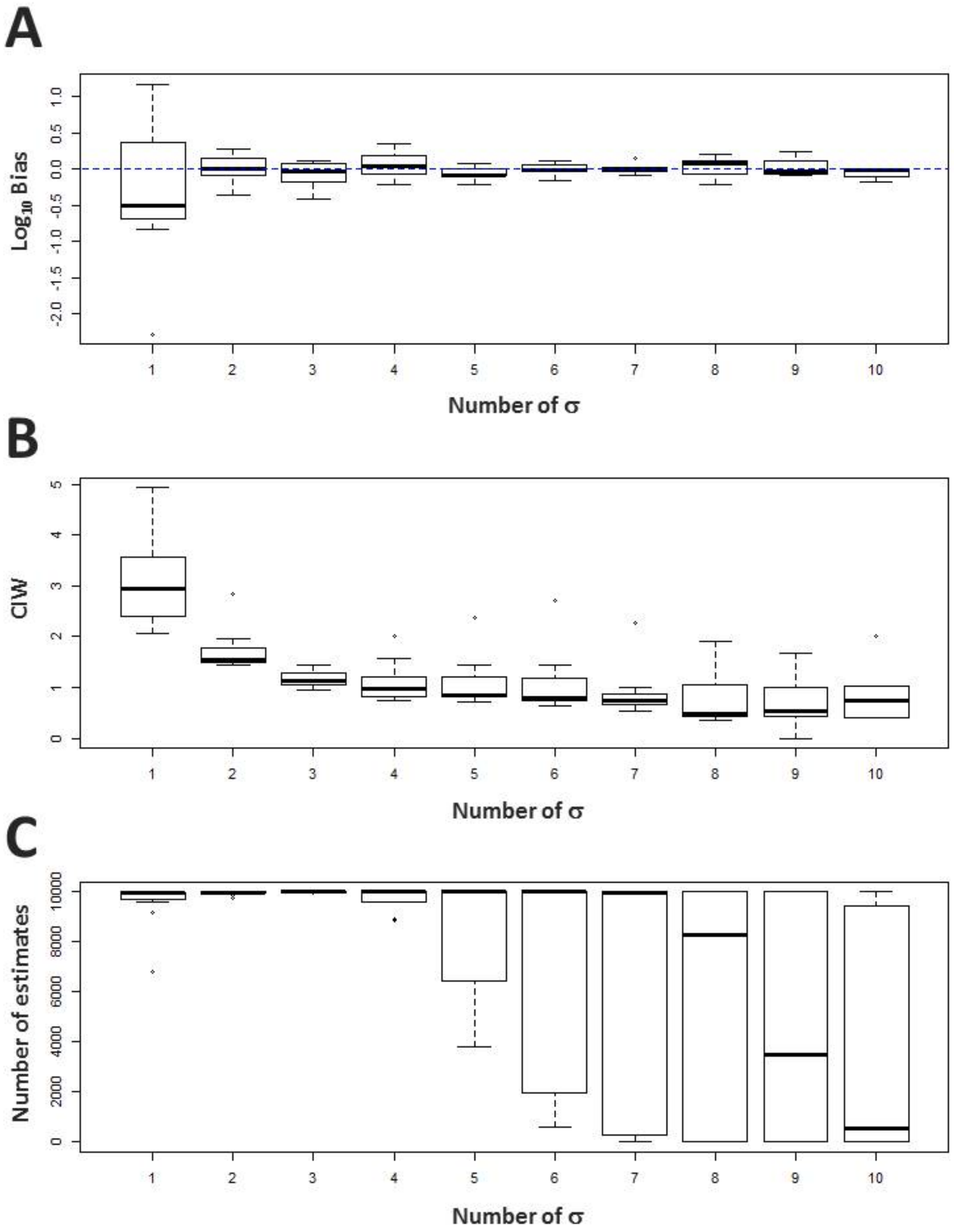

**Supplemental Figure S4.** Derivatives of the weight entropy score or measured assay response are calculated along the concentration grid. The vector of distances **x** = ($x_0$, …, $x_k$) are used for extrapolating $U_i$ or finding *nth* derivative $\frac{d^n U_0}{dC^n}$, where $|L|$ is the difference between the concentration at the origin of the grid and the first grid point evaluated. Specifically, the first derivatives require the terms $L + \alpha$, second derivatives require $L + \alpha + \beta$, third derivatives require $L + \alpha + \beta + \gamma$, fourth derivatives require $L + \alpha + \beta + \gamma + \delta$ and fifth derivatives require $L + \alpha + \beta + \gamma + \delta + \varepsilon$. The origin of the grid for the finite difference approach is shown as an open circle while the concentration grid points relative to the origin are shown as filled circles. The finite difference grid scheme is selected based on the derivative and the concentration to be calculated (see Table S1).

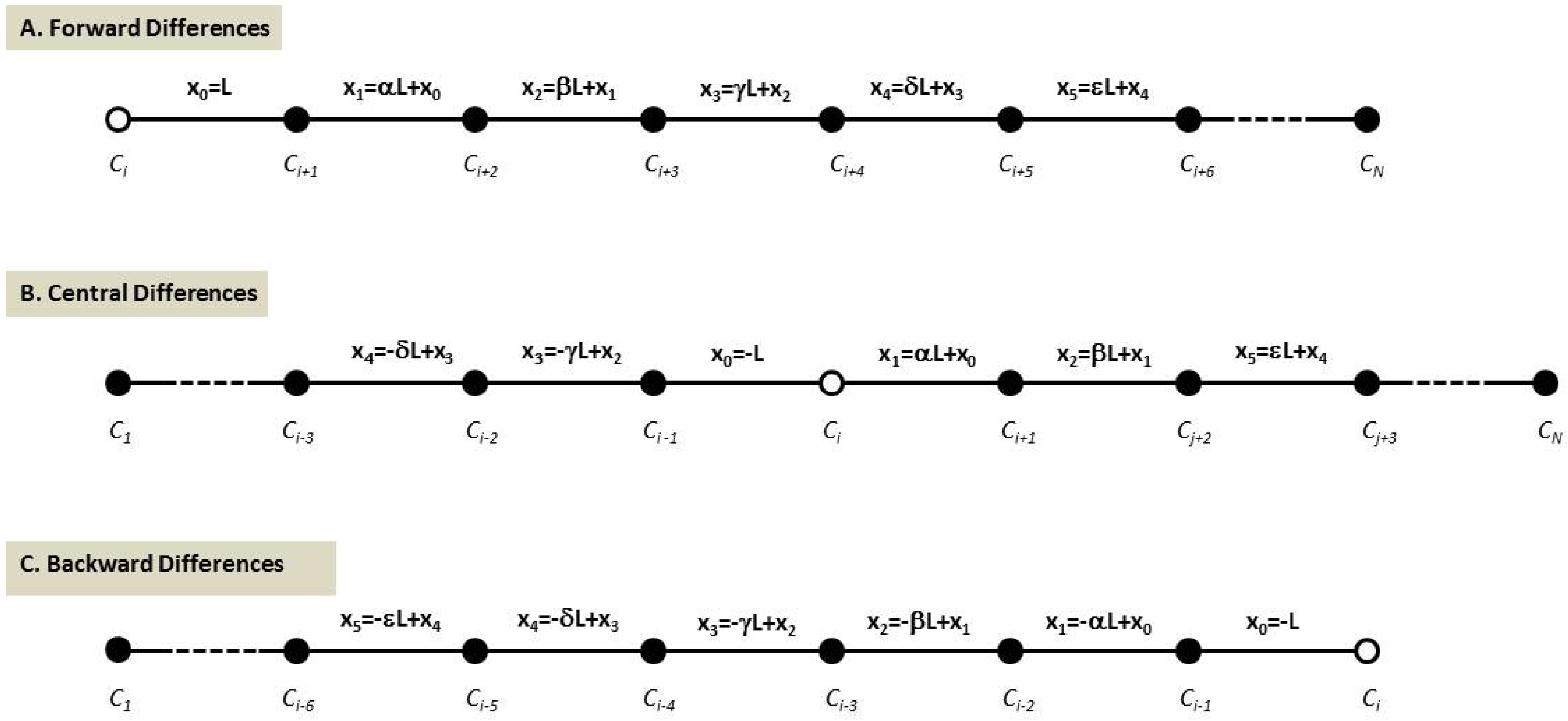

**Table S1. Finite Difference Selection Scheme for Leading Error on the Order of $L^2$**

| Derivative | Forward Differences | Central Differences | Backward Differences |
|---|---|---|---|
| First Derivative | conc = $C_1$ | $C_1$ < conc < $C_{N-1}$ | $C_{N-1}$ ≤ conc |
| Second Derivative | conc = $C_1$ | $C_1$ < conc < $C_{N-1}$ | $C_{N-1}$ ≤ conc |
| Third Derivative | conc ≤ $C_2$ | $C_2$ < conc < $C_{N-2}$ | $C_{N-2}$ ≤ conc |
| Fourth Derivative | conc ≤ $C_3$ | $C_3$ < conc < $C_{N-2}$ | $C_{N-2}$ ≤ conc |
| Fifth Derivative | conc ≤ $C_4$ | $C_4$ < conc < $C_{N-3}$ | $C_{N-3}$ ≤ conc |

**Supplemental References**


1. Lynch, D. R. *Numerical partial differential equations for environmental scientists and engineers*. (Springer, 2005).
2. Shockley, K. R. Using weighted entropy to rank chemicals in quantitative high-throughput screening experiments. *J Biomol Screen* **19**, 344-353 (2014).
3. R: A language and environment for statistical computing (R Foundation for Statistical Computing, Vienna, Austria, 2012).
4. Epperson, J. F. On the Runge example. *Amer Math Monthly* **94**, 329-341 (1987).
5. Press, W. H., Teukolsky, S.A., Vetterling, W.T., Flannery, B.P. *Numerical recipes: the art of scientific computing*. (Cambridge University Press, 2007).
6. Marsden, J., Weinstein, A. *Calculus I*. (Springer-Verlag New York Inc., 1985).
